# Syringomyelia in an Adult American Paint Horse

**DOI:** 10.3390/vetsci5020039

**Published:** 2018-04-05

**Authors:** Jacqueline P. Kurz, Kate E. Schoenhals, Gordon A. Hullinger, Arnaud J. Van Wettere

**Affiliations:** 1 School of Veterinary Medicine, Department of Animal, Dairy, and Veterinary Sciences, Utah State University, Logan, UT 84341, USA; jacqueline.larose@usu.edu; 2 Utah Veterinary Diagnostic Laboratory, Logan, UT 84341, USA; gordon.hullinger@usu.edu; 3 South Mountain Equine Veterinary Services, Bluffdale, UT 84065, USA; kate@southmountainequine.net

**Keywords:** syringomyelia, myelodysplasia, spinal cord, neurologic deficits, American Paint Horse

## Abstract

Syringomyelia is a form of myelodysplasia defined by the formation of one or more fluid-filled cavities within the spinal cord that do not communicate with the central canal. The defect may be congenital or acquired. Clinical signs correlate to the segment of spinal cord affected and include pain, paresis, proprioceptive deficits, alterations in sensation, scoliosis, and autonomic dysfunction. This report describes the clinical and pathologic changes in a case of acquired syringomyelia in a 10-year-old American Paint Horse mare. The horse had a six-week history of progressive proprioceptive deficits in all four limbs, bilateral pelvic limb ataxia, and muscle fasciculations that were unresponsive to treatment with stall rest, phenylbutazone, and dexamethasone. Syringomyelia was diagnosed postmortem within cervical, thoracic, and lumbar spinal cord segments. Acquired syringomyelia should be considered among differential diagnoses in adult horses displaying progressive neurologic deficits.

## 1. Introduction

Syringomyelia, a form of myelodysplasia, is the presence of tubular, fluid-filled cavities (syrinxes) within the spinal cord. The syrinxes are lined by glial cells and are filled by fluid that resembles normal cerebrospinal fluid. In contrast to hydromyelia, the cavities do not communicate with the central canal. Syringomyelia is uncommon in animals but has been reported in a number of species, including horses, cattle, dogs, and cats.

The defect may be congenital or acquired. Congenital syringomyelia may be a developmental anomaly, may be familial, or may be associated with infection by certain infectious agents. A familial form is reported in Weimaraner dogs [1]. Examples of syringomyelia associated with infectious agents have not been reported in horses, and are rarely reported in other species. Congenital syringomyelia and hydrocephalus associated with infectious agents include a cat infected with feline parvovirus [2] and a calf infected in-utero with bovine viral diarrhea virus [3]. Acquired syringomyelia occurs as a result of interference with normal drainage and/or volume of cerebrospinal fluid [4], which may be associated with the presence of space-occupying lesions, inflammation, trauma, or anatomical defects such as spinal cord tethering [5] or Chiari-like malformations [6]. Acquired syringomyelia has additionally been associated with ischemic loss of spinal cord tissue such as that occurring in thrombotic disease [5].

Clinical signs seen with syringomyelia are associated with the specific spinal segment(s) affected by the lesion, and result from loss and/or compression of nerve tracts. Clinical signs can include pain, paresis, proprioceptive deficits, ataxia, alterations in sensation, and autonomic dysfunction [4,7,8]. Clinical signs may be progressive as a result of expansion of the syrinxes over time.

The clinical, postmortem, and histopathologic findings of an adult horse diagnosed with syringomyelia are reported here. The onset of clinical signs in an adult horse suggests an acquired, rather than congenital, etiology. Syringomyelia has been reported in horses only rarely, but should be considered in horses with progressive neurologic deficits in which other causes have been ruled out.

## 2. Materials and Methods

A 10-year-old American Paint Horse mare presented to the South Mountain Equine Veterinary Services for investigation of lameness and peri-articular edema of the fetlock of the right pelvic limb. A cause was not identified clinically. Following worsening of clinical signs and the development of progressive neurologic deficits that were unresponsive to treatment, the mare was euthanized. Postmortem examination and histopathology were performed at the Utah Veterinary Diagnostic Laboratory. Gross lesions were identified and described, and fresh tissues were placed in 10% neutral buffered formalin for fixation. Central nervous system tissues were fixed prior to sectioning for gross examination. At least one cross-section and one longitudinal section of the spinal cord at each nerve root were trimmed in for histopathology. Tissues were routinely processed for histopathology, sectioned with a microtome at 5 µm, and stained with hematoxylin and eosin (H&E).

## 3. Results

### 3.1. Signalment, History, and Clinical Assessment

A 10-year-old, 483.5 kg, American Paint Horse mare presented with 4/5 (deLahunta Neurologic Scale), lameness and moderate peri-articular edema of the fetlock of the right pelvic limb. Radiographs of the fetlock of the right pelvic limb showed no bony changes or irregularities. Re-examination five days later revealed a marked improvement in the lameness but the presence of proprioceptive deficits in all four limbs. The lameness was attributed to trauma secondary to possible neurologic dysfunction. Stall rest was continued with intermittent turnout at the owner’s discretion.

One month later, the mare was re-examined and found to be alert and responsive but easily agitated. Her standing posture was abnormal, with all four legs placed base-narrow and adducted underneath the body center. Gait abnormalities were observed at a walk, and were particularly exaggerated in tight circles as well as over uneven ground surfaces (Grade 4/5 deLahunta Neurologic Scale). Due to the mare’s fractious disposition, grading of individual limb lameness was not performed. Ataxia and muscle weakness appeared more marked in the pelvic limbs, as demonstrated by the mare’s inability to elevate her pelvic limbs one at a time, especially her right pelvic limb. When walking, the thoracic limbs were stiff and hypermetric, resulting in stumbling. Bilateral muscle fasciculations were present over the shoulders, but muscle atrophy was not apparent. Cranial nerves and perineal reflexes were normal. No abnormalities or consistent indications of discomfort were apparent on palpation or manipulation. Clinical anatomic diagnosis indicated a focal or diffuse spinal cord lesion between C1 and C5 segments, with upper motor neuron and proprioceptive dysfunction present in all four limbs. One day later, the mare was unable to bear full weight on her left pelvic limb. Complete blood count parameters were within normal limits. Results of indirect fluorescent antibody testing were negative for *Sarcocystis neurona* and *Neospora hughesi* (University of California at Davis Veterinary Diagnostic Laboratory).

There was no clinical improvement over the next two weeks. The horse became increasingly agitated, anorexic and dangerous to caretakers and handlers. As a result, the horse was euthanized six weeks after initial presentation.

### 3.2. Treatments

Initial treatment for the injury to the fetlock of the right pelvic limb consisted of stall rest, a cold compress, and 2 g phenylbutazone (Vetribute; VetOne, Bimeda-MTC Animal Health, Inc., Cambridge, ON, Canada) per os. q. 24 h for seven days. Treatment following detection of neurological signs consisted of stall rest and 40 mg dexamethasone sodium phosphate (dexamethasone-SP; VetOne) intravenously followed by 20 mg dexamethasone (Par Pharmaceutical Companies, Inc., Spring Valley, NY, USA) per os. q. 24 h for four days.

### 3.3. Postmortem and Histopathology Findings

**Figure 1:**
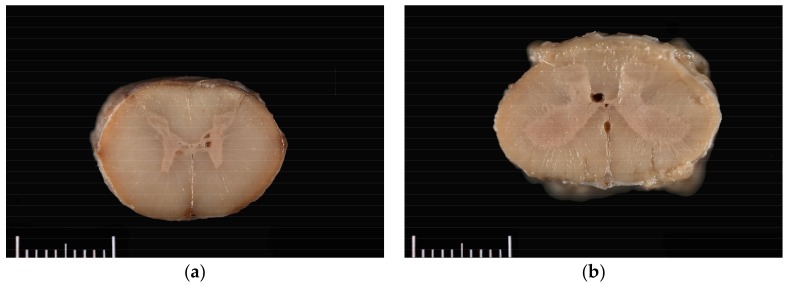
Grossly visible syrinxes within the grey commissures of the spinal cord at the level of (**a**) C5 and (**b**) L4.

The horse was necropsied at the Utah Veterinary Diagnostic Laboratory. The animal was in good body condition, with adequate musculature and modest subcutaneous and abdominal fat stores (body condition score 4.5/9). Multiple haemorrhages ranging in size from 1.0 × 1.0 × 0.5 cm to 10.0 × 10.0 × 0.5 cm were present in the subcutaneous tissue of the right dorsal and lateral hip, right lateral shoulder, and dorsal head between the pinnae. Upon trimming of the spinal cord after formalin fixation and at histologic evaluation, syrinxes were detected in the right and/or left grey commissures at C5–C6, T7, L1, and L4–L5; and in a lateral horn only at C5 and T14–T15. Syrinxes ranged in size from 125 µm to 2 mm diameter (L5) and consisted of non-communicating, empty cavities bordered by thin rims of compressed grey matter (Figure 1). No communication of the cavities with the central canal was detected grossly. No evidence of axonal degeneration, neuronal degeneration or necrosis, or inflammation was detected histopathologically.

## 4. Discussion

Syringomyelia has been reported previously in the literature in four horses, but only two of these were adults. In both adult cases, an acquired etiology was suspected. One report describes an eight-year-old thoroughbred stallion with syringomyelia secondary to a malignant neoplasm of the brainstem [9]. A separate report describes syringomyelia in a four-year-old pony. Although no additional lesions attributable to trauma were detected in the pony, trauma was considered possible [10].

In the horse reported here, a cause of acquired syringomyelia was not apparent. Past trauma is suspected as there was no evidence of neoplasia or infectious disease. Though lesions of past trauma were not evident at the time of death, resolution of trauma-induced lesions in other tissues is possible and considered more likely than resolution of lesions caused by other disease processes such as neoplasia. The subcutaneous haemorrhages noted at necropsy are considered to be evidence of trauma as a result of, rather than preceding, neurologic dysfunction associated with syringomyelia. Congenital syringomyelia cannot be entirely ruled out, but the age at onset of the clinical signs (10 years) makes this etiology less likely unless the condition was exacerbated by secondary factors.

Regardless of the inciting cause, obstruction of cerebrospinal fluid (CSF) flow is required for the development of syringomyelia. In acquired, posttraumatic syringomyelia, the initial spinal cord cavitation can be caused by a variety of factors, including mechanical damage due to cord compression, release of intracellular lysosomal or excitatory factors, liquefaction of intraparenchymal hematomas, and ischemia due to direct vascular damage or spinal cord tethering (subarachnoid adhesions as a result of trauma-induced arachnoiditis). Cavitation induced by any of these factors can influence disruption of CSF flow. For example, spinal cord tethering results in turbulent flow of CSF within the subarachnoid space, as the presence of adhesions disrupts the normal movement of CSF during the cardiac cycle [11]. Portions of the spinal cord are then subjected to abnormal pressure from CSF within the subarachnoid space, contributing to initial cavitation as well as expansion of syrinxes [6]. Clinical signs may be exacerbated during movement, excitement, or increased intra-abdominal pressure [4,9], when CSF pressure gradients are increased.

Clinical signs noted in this horse correspond to the spinal cord segments affected by syringomyelia. Proprioceptive deficits in all limbs, with abnormal gait and posture, are consistent with lesions noted at C5–C6. The syrinxes within this segment appear to disrupt the dorsal nucleus of Clarke, a cluster of sensory neurons involved in proprioception [12]. Lesions at T7 and T14–L1 may also have contributed to pelvic limb proprioceptive deficits via disruption and compression of the gracile fasciculus, the functions of which include relaying proprioceptive and sensory inputs [13]. Lesions at C5–C6 could contribute to sensory deficits in all four limbs, while the remaining lesions could contribute to sensory deficits in the pelvic limbs. Paresis, altered muscle tone, and altered limb reflexes could result from disruption of upper motor neuron tracts and inter-neurons. Upper motor neuron tracts in all limbs are expected to be disrupted as a result of lesions at C5, and upper motor neuron tracts in the pelvic limbs only as a result of lesions at T15–L1.

Apart from syringomyelia, no gross or microscopic evidence of metabolic, inflammatory, or structural abnormalities was found to account for the progressive fractiousness of the mare over the clinical course of disease. Therefore, the authors attribute the mare’s apparent agitation to pain secondary to syringomyelia.

Clinical signs in horses with syringomyelia reported previously vary, ranging from none to marked signs. In an adult stallion with acquired syringomyelia secondary to a brainstem neoplasm, syringomyelia was considered to be an incidental finding, with no clinical signs attributed to the presence of the syrinxes [10]. Clinical signs reported in other horses include proprioceptive deficits, scoliosis, limb spasticity, tremors, and altered sensation with self-mutilation [9,14].

In small animals, advanced imaging methods (magnetic resonance imaging (MRI), and computed tomography (CT) are valuable methodologies in the clinical diagnosis of syringomyelia. However, these imaging modalities are seldom available to large animal practitioners, and ante-mortem diagnosis can be problematic. In the case discussed here, transport of the mare to a referral diagnostic imaging center was considered unsuitable due to the severity of neurologic deficits, which would have compromised the mare’s welfare during transport. In a large animal medicine setting, specialized diagnostic imaging may also be cost-prohibitive or logistically impractical in many cases. Diagnosis of syringomyelia in large animals often requires postmortem examination for definitive diagnosis.

## 5. Conclusions

Although syringomyelia appears to be rare in horses, this report indicates that syringomyelia should be considered among differential diagnoses in horses with proprioceptive deficits, particularly when a history of past trauma is suspected.
